# Elevated IGFBP4 and Cognitive Impairment in a PTFE-Induced Mouse Model of Obstructive Sleep Apnea

**DOI:** 10.3390/ijms26157423

**Published:** 2025-08-01

**Authors:** E. AlShawaf, N. Abukhalaf, Y. AlSanae, I. Al khairi, Abdullah T. AlSabagh, M. Alonaizi, A. Al Madhoun, A. Alterki, M. Abu-Farha, F. Al-Mulla, J. Abubaker

**Affiliations:** 1 Biochemistry & Molecular Biology, Dasman Diabetes Institute, Dasman 15462, Kuwait; yara.alsanae@dasmaninstitute.org (Y.A.); irina.alkhairi@dasmaninstitute.org (I.A.k.); mohamed.abufarha@dasmaninstitute.org (M.A.-F.); 2 Animal & Imaging Facility, Dasman Diabetes Institute, Dasman 15462, Kuwait; nermeen.abukhalaf@dasmaninstitute.org (N.A.); ashraf.madhoun@dasmaninstitute.org (A.A.M.); 3 Department of Anatomy, College of Medicine, Health Sciences Center, Kuwait University, P.O. Box 24923, Safat 13110, Kuwait; a.alsabagh@ku.edu.kw (A.T.A.); mohammed.alonaizi@dasmaninstitute.org (M.A.); 4 Kuwait Board of Post Graduate Training Program, Head & Neck Surgery Faculty, Kuwait Institute for Medical Specialisation (KIMS), P.O. Box 24923, Safat 13110, Kuwait; abdulmohsen.alterki@dasmaninstitute.org; 5 Department Otolaryngology, Head & Neck Surgery, Zain & Al Sabah Hospitals, Safat 13083, Kuwait; 6 Research Sector, Dasman Diabetes Institute, Dasman 15462, Kuwait; 7 Department of Translational Research, Dasman Diabetes Institute, Dasman 15462, Kuwait; fahd.almulla@dasmaninstitute.org

**Keywords:** obstructive sleep apnea, hypoxia, OSA animal model, IGFBP4, biomarker, open field test, novel object recognition

## Abstract

Obstructive sleep apnea (OSA) is a prevalent disorder linked to metabolic complications such as diabetes and cardiovascular disease. By fragmenting normal sleep architecture, OSA perturbs the growth hormone/insulin-like growth factor (GH/IGF) axis and alters circulating levels of IGF-binding proteins (IGFBPs). A prior clinical observation of elevated IGFBP4 in OSA patients motivated the present investigation in a controlled animal model. Building on the previously reported protocol, OSA was induced in male C57BL/6 mice (9–12 weeks old) through intralingual injection of polytetrafluoroethylene (PTFE), producing tongue hypertrophy, intermittent airway obstruction, and hypoxemia. After 8–10 weeks, the study assessed (1) hypoxia biomarkers—including HIF-1α and VEGF expression—and (2) neurobehavioral outcomes in anxiety and cognition using the open-field and novel object recognition tests. PTFE-treated mice exhibited a significant increase in circulating IGFBP4 versus both baseline and control groups. Hepatic Igfbp4 mRNA was also upregulated. Behaviorally, PTFE mice displayed heightened anxiety-like behavior and impaired novel object recognition, paralleling cognitive deficits reported in human OSA. These findings validate the PTFE-induced model as a tool for studying OSA-related hypoxia and neurocognitive dysfunction, and they underscore IGFBP4 as a promising biomarker and potential mediator of OSA’s systemic effects.

## 1. Introduction

Obstructive sleep apnea (OSA) is a widespread sleep-related condition characterized by recurrent upper airway obstructions during sleep, leading to intermittent hypoxia (IH), hypercapnia, hypoxemia, and fragmented sleep [1]. Epidemiological studies reported that 24% of men and 9% of women in the adult population (30–60 years) are diagnosed with OSA [2]. People with obesity and advancing age are at a higher risk of developing OSA, while the prevalence rises with additional risk factors like ethnicity and harmful lifestyle habits [3]. One of the main OSA risk factors is obesity, whereby a higher body mass index (BMI) shows a linear positive correlation with OSA [4]. This link suggests that nations with elevated obesity rates would have higher prevalence and incidence rates of OSA.

Additionally, an independent risk factor for OSA is male sex, which increases the estimated male-to-female prevalence ratio to 1.5:1; nonetheless, the underlying reasons remain elusive [5]. Epidemiological reports have shown that OSA is positively linked to an increased risk of cardiovascular disease [6], metabolic dysfunction [7], and neurocognitive impairment [8,9,10]. OSA has also been found to contribute to the development of nonalcoholic fatty liver disease [11]. Recent reports provide evidence for the involvement of OSA in a range of diseases, including hypertension [12], glucose metabolism [13], and insulin resistance [14]. However, a comprehensive understanding of the pathogenic mechanisms has yet to be formulated. Although progress has been made in investigating pathophysiological mechanisms and therapeutic interventions for OSA, the pace has been relatively slow due to various challenges and limitations of disease models. Thus, OSA animal models have opened the door for replicating human-like conditions [15].

Animal models, especially rodents, have provided valuable insights into the systemic impact of OSA. The chronic intermittent hypoxia (CIH) model is widely used to mimic the hypoxic–reoxygenation cycles in OSA. Studies employing CIH in rodent models have demonstrated its association with adverse outcomes such as hypertension [16], insulin resistance [17], and endothelial dysfunction [18]. This model, with others like the upper airway collapse model [19], the New Zealand obese (NZO) mouse model [20], and the brachycephalic obstruction airway syndrome (BOAS) model [21], has been critical in understanding the systemic effects of intermittent hypoxia. However, they often fail to replicate the mechanical airway obstructions intrinsic to OSA [15]. Other researchers have utilized OSA modeling by introducing techniques such as nerve stimulation-induced airway collapse [10,22,23], pharyngeal narrowing/occlusion during central sleep apnea, and genetic modification models to study susceptibility to hypoxia [24]. These approaches provide deeper insight into the complex interactions between airway mechanics and hypoxic stress.

To overcome such limitations, newer animal models have been developed to simulate spontaneous airway obstruction during sleep. Using the polytetrafluoroethylene (PTFE) injection method in C57BL/6 mice led to tongue enlargement, which mimicked human-like airway obstruction [25]. Lebek et al. demonstrated that this approach replicates spontaneous apneas and highlights the associated cardiovascular implications, such as left ventricular contractile dysfunction [25]. To investigate potential biomarkers for hypoxia and OSA, we utilized the spontaneous upper airway narrowing model in our study. We validated our model by examining the impact on anxiety and cognitive function and evaluated changes in specific hypoxia biomarkers. Identifying novel biomarkers linked to OSA would support early diagnosis, which would help avoid and prevent the harmful complications associated with OSA.

The IGFBP family includes high-affinity binding proteins (IGFBP1–IGFBP6) [26] and IGFBP7 that shares up to 45% similarity to other IGFBPs [27]. This family of proteins is critical for regulating the GH/IGF system and IGF ligand signaling and bioavailability [26]. The GH/IGF and IGFBPs system was found to be affected by sleep and disturbed sleep patterns [28], suggesting a potential involvement for IGFBPs in OSA or related processes. IGFBP family members participate in cell proliferation, differentiation, migration, and apoptosis, which contributes to healthy and pathological processes [26,29]. The hepatic IGFBP1 plays a role in insulin sensitivity, glucose control, and cardiovascular pathology [30,31]. Under extreme conditions of hypoxia and hunger, levels of IGFBP1 are elevated to inhibit IGF activity and growth [29]. Alternatively, IGFBP3 is primarily associated with regulating cell development and death [32], and reduced IGFBP3 levels are linked to increased CVD risk and mortality [33,34]. Additionally, children with OSA were found to have low IGFBP3 levels, which was linked to growth retardation, thus underscoring its importance [35]. Furthermore, the AHI score, which measures the severity of sleep apnea, was found to correlate negatively with IGF-I levels, regardless of BMI or age [28,36]. In a previous report involving patients with OSA, we reported a significant rise in levels of circulating IGFBP4 that showed a substantial drop following a treatment intervention [37].

Through this work, we intend to enhance the current understanding of OSA by using a mouse model that closely resembles spontaneous upper airway obstruction and OSA pathology in humans. The model is primarily verified by detecting changes in known hypoxia markers associated with OSA and by assessing changes in the animals’ cognitive function and anxiety-like behavior. By achieving these goals, this study aims to present a commodious tool that can be used to illuminate the underlying processes of OSA and its systemic implications, ultimately leading to improved diagnostic and treatment options. Our findings will shed light on potential biomarkers for the early detection and prevention of OSA-related complications.

## 2. Results

### 2.1. The Effect of Hypoxia on IGFBP4 Expression In Vitro

**Figure 1:**
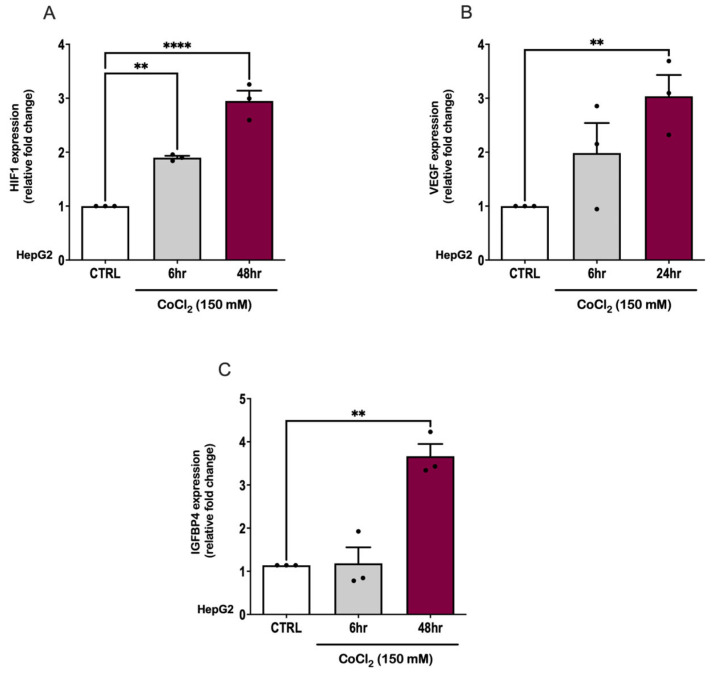
Hypoxia induction by CoCl_2_, a hypoxia mimetic agent, indicated by hypoxia markers in HepG2 and the concurrent increase in IGFBP4 expression. (**A**) HepG2 cells showing an increase in HIF1α expression after 6 h CoCl_2_ treatment (mean = 1.89 ± 0.033, *p* = 0.015) that peaked after 48 h treatment (mean = 2.92 ± 0.19, *p* < 0.0001) compared with the CTRL. (**B**) HepG2 cells showed a significant increase in VEGF expression due to CoCl_2_ treatment after 24 h (mean = 3.04 ± 0.39, *p* = 0.007) compared with the CTRL. (**C**) CoCl_2_-induced hypoxia in HepG2 increased the expression of the IGFBP4 gene (mean = 3.67 ± 0.48, *p* = 0.003). A groups comparison was performed using a one-way ANOVA test, and a post hoc Dunnett test was used to correct for multiple comparisons. Statistical significance was determined with ** *p*-values < 0.01 and **** *p*-values < 0.0001.

In a previous study, we reported a significant rise in IGFBP4 levels under conditions of hypoxia in a cohort diagnosed with obesity and OSA [37]. To explore IGFBP4 regulation by hypoxia in an in vitro setup, we employed HepG2 cells that were incubated under hypoxic conditions. Hypoxia was induced chemically by treating HepG2 cells with a hypoxia mimetic agent, CoCl_2_ (150 μM), for 6 to 48 h. This treatment showed a significant 4-fold increase in IGFBP4 gene expression that concurred with a 3-fold elevation in HIF1α and VEGF gene expression, confirming the induction of hypoxia (Figure 1).

### 2.2. In Vivo Study—Tongue Enlargement Using a Bulking Agent

**Figure 2:**
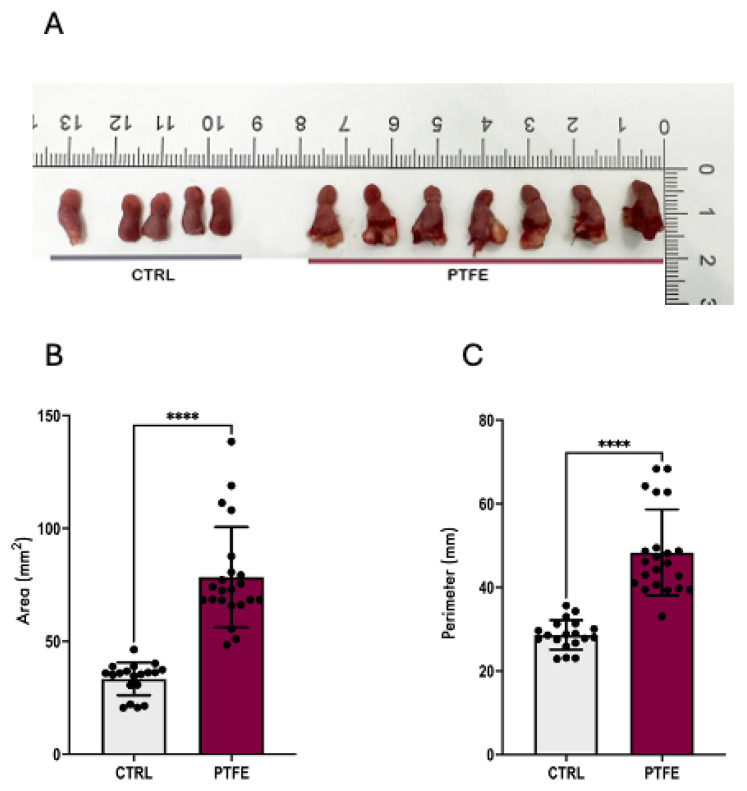
PTFE-injected tongue(s) expand significantly compared with CTRL animals. (**A**) Sample of extracted tongues from sacrificed animals after an 8-week follow-up period, comparing PTFE-injected mice with CTRL mice (glycerol-injected). (**B**) Quantification of the total tongue area shows a significant increase in the total tongue area *p*-value < 0.0001 of the PTFE-injected group (mean = 78.41 ± 1.6 mm^2^) compared with the CTRL (mean = 33.4 ± 4.7 mm^2^). (**C**) Tongue perimeter measurement shows a significant increase in the PTFE group (mean = 28.64 ± 0.81 mm) compared with the CTRL (mean = 48.34 ± 2.1 mm). Unpaired Student’s *t*-test was used to test statistical significance between PTFE and CTRL groups, with **** *p*-values < 0.0001 indicating significance.

Injecting the tongue of lean male C57BL/6 mice with polytetrafluoroethylene (PTFE) caused a significant 1.6-fold increase in the tongue diameter relative to CTRL animals (Figure 2B,C). PTFE-injected animals showed an increase in the total tongue area (mean = 78.41 ± 1.6 mm^2^, *p*-value < 0.0001) compared with the CTRL (mean = 33.4 ± 4.7 mm^2^, Figure 2B). Tongue enlargement was evident after 2 weeks of PTFE injection, and PTFE polymer depots remained in the tongue for the whole follow-up period, causing a permanent tongue expansion (Figure 2A). After sacrificing the animals, we measured tongue diameter and thickness, which showed a significant and apparent difference in the tongue size and shape, comparing PTFE-injected mice with CTRL mice (Figure 2B,C).

### 2.3. Daily Activity and Behavior

**Figure 3:**
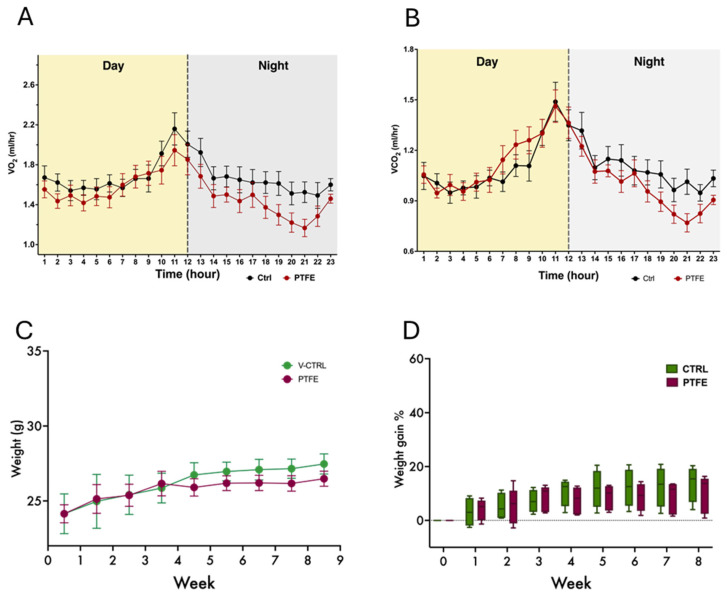
PTFE-induced tongue expansion affects the exchange of gases. (**A**) A 24-h representative plot of oxygen consumption (VO2) shows a difference between the PTFE group (red) and the CTRL (black) in the amount of oxygen consumed, particularly during the dark cycle. (**B**) A representative plot showing a real-time recording of carbon dioxide production, with data compiled from PTFE (red) and CTRL (black). (**C**) Weight change and normal growth progression throughout the experiment period in all groups, with no significant difference between the groups. (**D**) Calculated percentage of weight gain, showing the mean percentage of weight gain throughout the study period. Weight gain percentage across groups was comparable, showing no significant difference between PTFE and CTRL.

The animals were housed in metabolic cages two weeks after the PTFE intervention to monitor their daily metabolic activity, general behavior, and breathing/gas exchange patterns. Our data reflected comparable activity from both groups, PTFE and CTRL; however, it showed some differences in their respiratory functions, reflected by the gas exchange data (Figure 3A,B). Additionally, this intervention is expected to disturb the ability of animals to consume food normally; therefore, we monitored changes in the animals’ total body weight of animals throughout the experimental period. Although tongue intervention caused initial discomfort for the animals, our data showed insignificant differences in the gradual changes in weight gain throughout the follow-up 8-week period (Figure 3C,D).

### 2.4. PTFE-Treated Animals Display Anxiety-like Behavior and Memory Impairment

**Figure 4:**
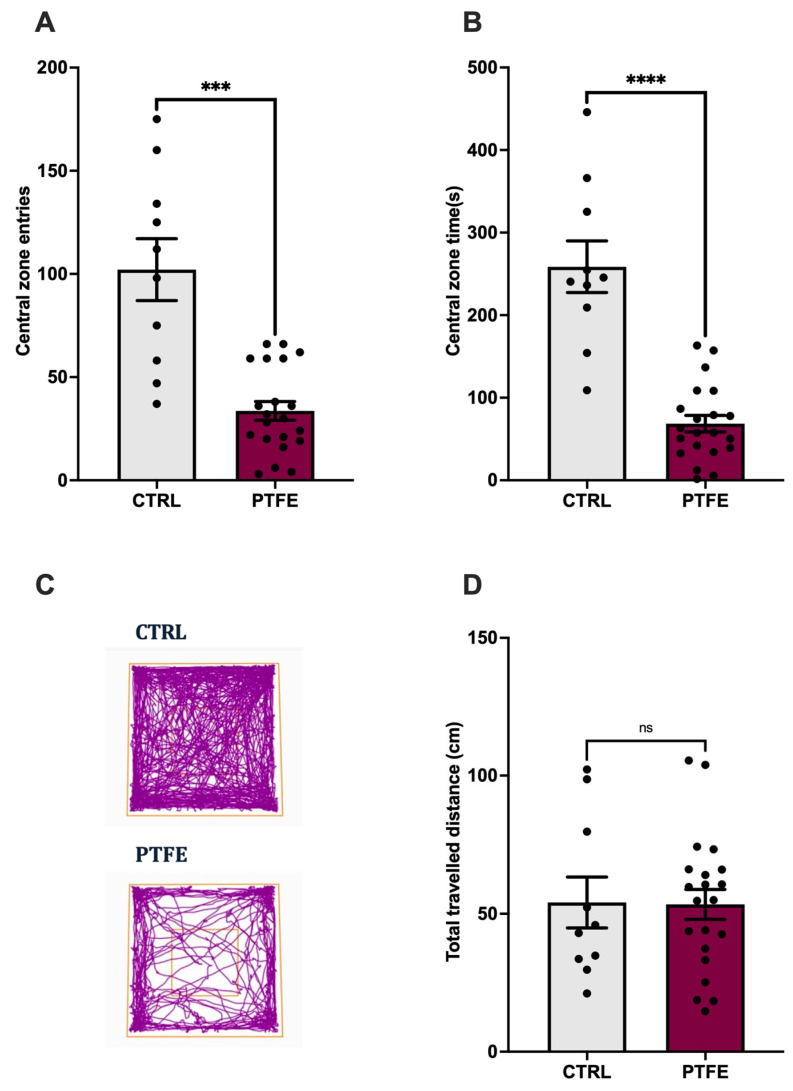
PTFE treatment induces anxiety-like behavior in animals after 8 weeks of intervention, as shown by the open-field test. (**A**) PTFE mice made significantly fewer entries to the central zone (18.68 ± 2.5 entry, *p* < 0.0001, n = 21) than CTRL mice (entry attempt = 56.7 ± 8.34, n = 10). (**B**) The PTFE group spent significantly less time (mean = 68.53 ± 9.9, *p* < 0.0001, n = 21) in the central zone than the CTRL (mean = 258.7 s ± 31.23, n = 10). (**C**) Representative activity trace plots of CTRL animals compared with PTFE. (**D**) The total distance traveled shows no difference between CTRL and PTFE (mean = 77.21 cm ± 7.5, mean = 53.41 ± 5.4, respectively). An unpaired Student’s *t*-test was used to test statistical significance between PTFE and CTRL groups, with *** *p*-values < 0.001 and **** *p*-values < 0.0001 indicating significance.

To understand the impact of PTFE-treatment on animals, we explored specific cognition domains that reflect anxiety and depression, as well as learning and memory functions. We evaluated the anxiety level by performing the open-field test (OFT) to analyze the animal activity level in the central zone [38,39]. Our data showed that animals from the PTFE group made significantly fewer entries to the central zone (entry attempt mean = 18.68 ± 2.5, n = 21) compared with the CTRL group (entry attempt mean = 56.7 ± 8.34, n = 10, Figure 4A). Additionally, they spent less time (PTFE = 4.2 ± 0.64 s, n = 21) in the central zone compared with the CTRL (mean = 14.4 ± 1.7 s, n = 10), as shown in Figure 4B. This reflected an apparent anxious-like behavior, as depicted from the representative activity trace plots for CTRL and PTFE animals (Figure 4C). Interestingly, our data showed that the total distance traveled by animals in both groups was comparable with no significant difference (PTFE = 60.88 ± 5.9 cm and CTRL = 59.71 ± 10.05 cm, Figure 4D).

**Figure 5:**
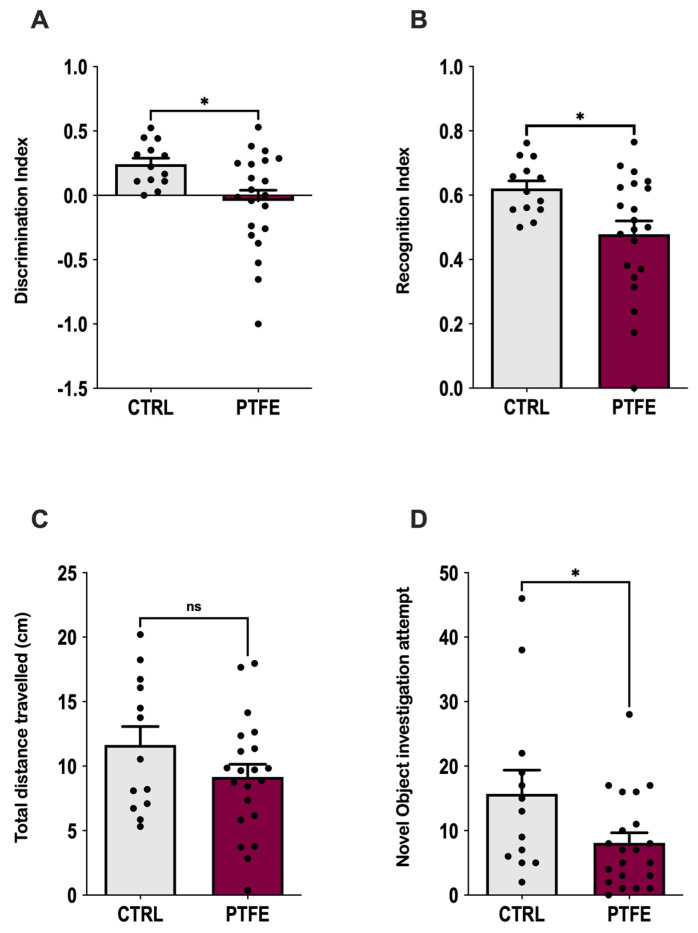
Exploring the effect of PTFE treatment on memory and learning through the novel object recognition test. (**A**) Discrimination index of PTFE and CTRL mice in the testing phase, showing a significantly reduced cognitive ability in the PTFE group (mean = −0.043 ± 0.083, *p* = 0.016, n = 21) compared with the CTRL (mean = 0.24 ± 0.046, n = 13). (**B**) Recognition index in the testing phase, showing a significantly lower value in the PTFE group (mean = 0.478 ± 0.041, *p* = 0.0161, n = 21) compared with the CTRL (mean = 0.62 ± 0.023, n = 13). (**C**) Total distance traveled by animals from both groups is comparable with no significant difference (CTRL = 11.6 ± 1.4, PTFE = 9.15 ± 0.98, *p* = 0.15). (**D**) Novel object investigation attempts by the PTFE group (mean = 8.09 ± 1.5 times) were significantly less than attempts by the CTRL (mean = 15.69 ± 3.7, *p* = 0.037). An unpaired Student’s *t*-test was used to test statistical significance between PTFE and CTRL groups, with * *p*-values < 0.05 indicating significance.

Another aspect of cognitive functions is testing the animal’s learning ability and memory, which we explored using the novel object recognition test (NOR) [40,41]. Our data showed that animals from the PTFE group had impaired memory and learning ability compared with the CTRL (Figure 5). This was reflected by the fewer exploration attempts of the novel object by PTFE animals (mean = 8.09 ± 1.5 times) compared with the CTRL (mean = 15.69 ± 3.7 times, *p*-value = 0.037, Figure 5D). NOR results presented PTFE-treated animals with a significantly lower discrimination index value (mean = −0.043 ± 0.083, *p*-value = 0.016, n = 21) compared with animals from the CTRL group: mean = 0.24 ± 0.046, n = 13 (Figure 5A). Moreover, the recognition index showed a significantly lower value in the PTFE group (mean = 0.478 ± 0.041, *p*-value = 0.0161, n = 21) than CTRL = 0.62 ± 0.023, n = 13 (Figure 5B). This suggests an impaired cognitive ability after 8 weeks of PTFE treatment that was absent from the CTRL group. We found no significant difference in the total distance traveled by animals from both groups (CTRL = 11.6 ± 1.4 cm, PTFE = 9.15 ± 0.98 cm, *p*-value = 0.15, Figure 5C). This indicates that animals’ mobility and locomotion were not compromised.

### 2.5. PTFE-Injected Animals Show a Significant Increase in the Expression of Hypoxia Marker

**Figure 6:**
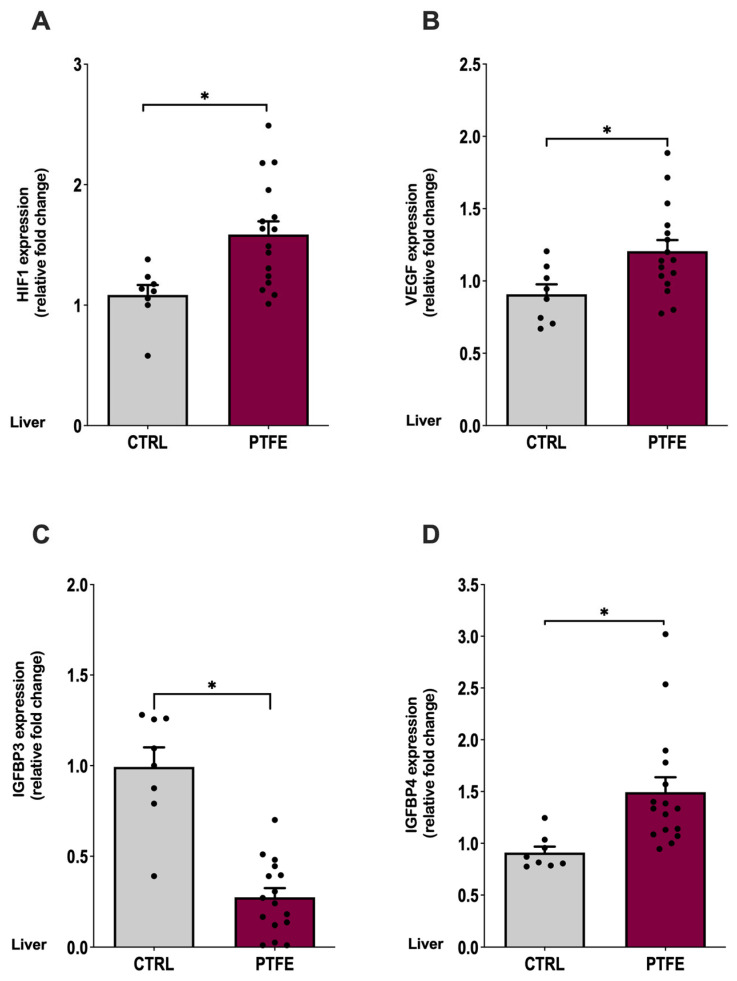
Intermittent hypoxia induces the expression of IGFBP4 at the gene level in the liver. Tongue-induced expansion increased the gene expression of hypoxia markers; (**A**) HIF1α in PTFE = 1.72 ± 0.28, *p* = 0.044 compared with CTRL = 1.08 ± 0.08. (**B**) VEGF expression level in PTFE = 1.24 ± 0.09, *p* = 0.042 compared with CTRL = 0.903 ± 0.08. (**C**) IGFBP3 gene expression significantly declined in PTFE = 0.29 ± 0.1, *p* = 0.025 compared with the CTRL = 0.98 ± 0.29. (**D**) IGFBP4 gene expression increased significantly in PTFE = 1.51 ± 0.15, *p* = 0.048 compared with CTRL = 0.93 ± 0.09. An unpaired Student’s *t*-test was used to test statistical significance between PTFE and CTRL groups, with * *p*-values < 0.05 indicating significance.

Hypoxia-inducible factor 1α (HIF1α) and vascular endothelial growth factor (VEGF) are important biomarkers indicating the presence of hypoxia [42,43]. We assessed HIF1α and VEGF mRNA expression in the liver tissue using qPCR. Our data revealed a significant increase in HIF1α expression in the PTFE group = 1.72 ± 0.28, *p* = 0.044, compared with CTRL = 1.08 ± 0.08 (Figure 6A). Another marker is VEGF, which is a well-recognized hypoxia biomarker that is induced by exposure to systemic hypoxia. Pathological conditions involving hypoxia were identified to stimulate VEGF induction [44]. Our data showed a significant rise in VEGF expression (mean = 1.24 ± 0.09, *p* = 0.042) compared with the CTRL (mean = 0.903 ± 0.08, Figure 6B). In addition to the classic hypoxia biomarkers, we examined the effect of PTFE injection on IGFBP4 as a novel biomarker previously reported for hypoxia/OSA [37].

Consistent with Alterki et al.’s [37] observation, we found a significant increase in IGFBP4 mRNA expression (PTFE = 1.51 ± 0.15, *p* = 0.048, Figure 6D) compared with the CTRL (mean = 0.92 ± 0.09). This indicates the effectiveness of PTFE intervention in mimicking OSA and inducing hypoxic events that elevated the expression of IGFBP4. Interestingly, IGFBP3 gene expression was significantly reduced (PTFE = 0.287 ± 0.102, *p* = 0.025) compared with the CTRL (mean = 0.98 ± 0.29).

**Figure 7:**
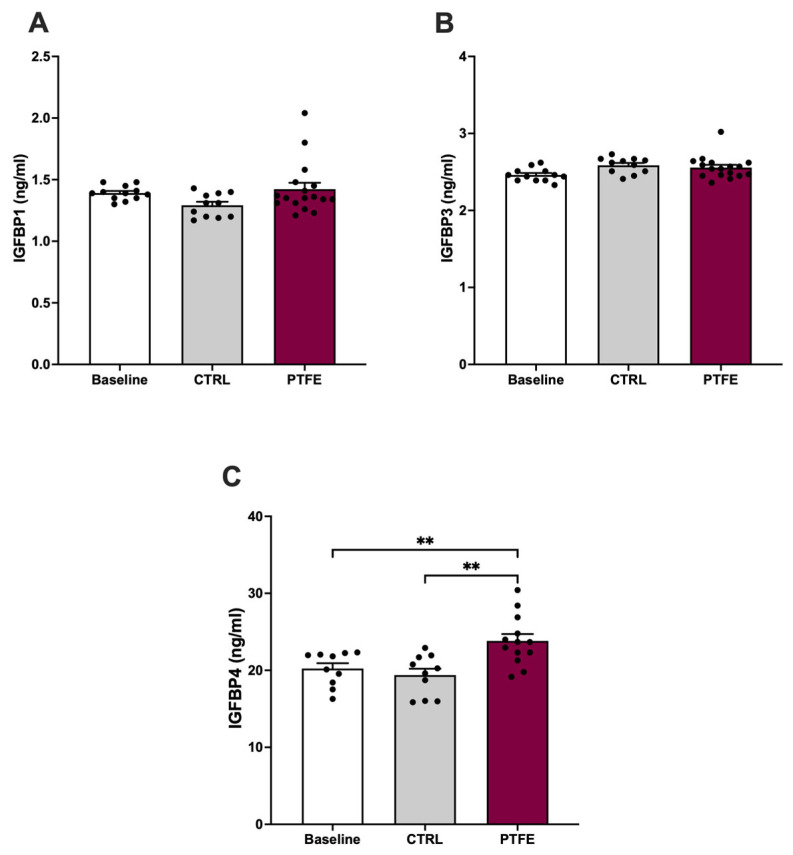
Significant increase in circulating IGFBP4 with intermittent hypoxia. To evaluate the effect of hypoxia on IGFBPs, we quantified circulation. (**A**) IGFPB1 showing no difference in plasma IGFBP1 levels between baseline (mean = 1.39 ± 0.02 ng/mL) and post-intervention (PTFE = 1.4 ± 0.07 ng/mL, CTRL = 1.34 ± 0.04 ng/mL). (**B**) No significant change in IGFBP3 levels before (mean = 2.4 ± 0.09 ng/mL) and after intervention (PTFE = 2.54 ± 0.03 ng/mL, CTRL = 2.58 ± 0.02 ng/mL). (**C**) Circulating IGFBP4 levels showed a significant increase in PTFE group (mean = 23.65 ± 0.67 ng/mL) compared with CTRL (mean = 19.55 ± 1.3 ng/mL) and baseline (mean = 20.2 ± 0.98 ng/mL). Groups comparison was performed with a one-way ANOVA test, and a post hoc Dunnett test was used to correct for multiple comparisons. Statistical significance was determined with ** *p*-values < 0.01.

Furthermore, we analyzed circulating biomarkers focusing on the IGFBP family. Blood samples were collected from animals 8 weeks post-PTFE injection. Our data showed that changes in circulating IGFBPs were specific and only affected certain proteins (Figure 7). We found no changes in circulating IGFBP1 (mean = 1.4 ± 0.07 ng/mL) and IGFPB3 (mean = 2.54 ± 0.03 ng/mL) compared with baseline (IGFBP1 =1.39 ± 0.02 ng/mL and IGFBP3 =2.4 ± 0.09 ng/mL) pre-PTFE treatment and compared with the control group (IGFBP1 = 1.34 ± 0.04 ng/mL and IGFBP3 = 2.58 ± 0.02 ng/mL) (Figure 7A,B). Although circulating levels of IGFBP1 and IGFBP3 did not show a change, we found a significant increase in IGFBP4 levels after 8 weeks of PTFE treatment (mean = 23.65 ± 0.67 ng/mL) compared with both the baseline (mean = 20.2 ± 0.98 ng/mL) and CTRL group (mean = 19.55 ± 1.3 ng/mL) (Figure 7C).

## 3. Discussion

In this report, we examined the efficacy of IGFBP4 as a prospective biomarker for obstructive sleep apnea utilizing the PTFE OSA mouse model, as outlined by Lebek et al. [25]. Our results indicate a substantial elevation in circulating IGFBP4 levels and hepatic IGFBP4 gene expression in mice exposed to PTFE-induced chronic intermittent hypoxia, simulating OSA conditions. These findings collectively support our previously reported finding of IGFBP4 as a biomarker for OSA and underscore its potential role in the pathophysiological pathways contributing to OSA. The significant increase in the expression of two recognized indicators of hypoxic stress, HIF1α and VEGF, supports the strength of the hypoxia-induced physiological alterations in our current model. Moreover, employing tests like the open-field and novel object recognition tests to evaluate behavioral changes indicates that exposure to chronic intermittent hypoxia detrimentally affected cognitive performance and elicited anxiety-like behaviors.

To properly utilize the OSA model, we applied several indirect yet sensitive methods to confirm hypoxia in the PTFE OSA mouse. Chronic intermittent hypoxia, a hallmark of OSA, is known to damage brain function through oxidative stress, neuroinflammation [45,46], and neuronal malfunction, leading to cognitive deficiencies and behavioral difficulties [47,48,49]. Repeated instances of hypoxemia and sleep disruption in OSA initiate a sequence of detrimental mechanisms leading to neuronal damage in brain areas essential for cognition, such as the hippocampus and prefrontal cortex [50,51]. These injuries hinder synaptic plasticity and compromise long-term potentiation, both of which are crucial for memory formation and learning [52]. The NOR test evaluates hippocampal-dependent memory [40], while the OFT assesses anxiety-like behaviors [53]. Previous studies have reported that exposure to hypoxia affects crucial processes and leads to diminished performance due to disturbances in synaptic plasticity and neurotransmitter equilibrium [45,49,54]. OSA also jeopardizes neuronal health by inducing endothelial injury and microvascular impairment and decreasing cerebral blood flow [55]. Moreover, sleep fragmentation disrupts sleep architecture by tempering sleep spindles and slow-wave activity during NREM sleep, which is crucial for memory consolidation and cognitive processing [55,56].

Our data showed a diminished exploration of the novel object after training in the NOR test, which signifies an impaired recognition memory. This was complemented with an elevated anxiety level that was reflected by the reduced entrance and locomotion into the central zone in the open-field test, which collectively aligns with a hypoxia-induced neurocognitive impairment [45]. These behavioral anomalies reflect the cognitive impairments normally seen in OSA patients, like executive dysfunction and memory deficiencies [57]. Studies examining OSA patients have also reported structural alterations in the brain, including grey matter reduction in the hippocampus and frontal cortex, which was correlated with cognitive impairment severity. Interestingly, some of these alterations were found to be reversible with the appropriate therapy [51,55]. Alongside evaluating changes to behavioral activity, validating molecular markers of hypoxia such as HIF1α and VEGF provides solid support for the hypoxia-induced effect in our animal model [58], thus enhancing the credibility of the used OSA animal model and its applicability as a model mimicking human OSA.

Among the IGFBP family, IGFBP4 protein is the smallest protein primarily secreted by the liver to regulate the biological effects of IGF-I and IGF-II [59]. Various growth factors, hormones, and IGFBP proteinases regulate IGFBP4 expression levels across different tissues [60]. Due to its regulatory role in the IGF signaling system, IGFBP4 has emerged as a potential biomarker in various pathological conditions, such as cancer [61], cardiovascular diseases [62], and metabolic disorders [63]. Nonetheless, its significance in the context of OSA is still relatively unexplored. Our data demonstrate a significant increase in circulating IGFBP4 and hepatic IGFBP4 gene expression levels, indicating that IGFBP4 is potentially susceptible to hypoxic stress. This in vivo data is corroborated by our in vitro studies, whereby CoCl_2_-induced hypoxia in HepG2 cells caused a significant upregulation of IGFBP4 gene expression (Figure 1C) and protein expression. This agrees with a previous study that has reported IGFBP4 upregulation under hypoxic conditions [64]. This finding is consistent with our earlier report, where people with OSA had a significant increase in circulating IGFBP4 [37].

In the context of angiogenesis and tumor growth, Igfbp4 gene expression was upregulated in response to hypoxia, which substantiates the role of Igfbp4 as a hypoxia-regulated gene with a potential role in controlling tumor growth [65]. Recently, Sun et al. found that IGFBP4 is significantly downregulated in metastatic hepatocellular carcinoma, implicating a protective role for IGFBP4 overexpression to restrain cancer metastasis and cell migration [66]. In a different context, Torres et al. demonstrated a substantial increase in serum IGFBP4 levels in people suffering from pulmonary arterial hypertension (PAH), whereby IGFBP4 was presented as a circulating prognostic biomarker and was linked to disease severity [67]. Additionally, elevated expression level of IGFBP4 was detected in the atherosclerotic plaque and blood samples from people with atherosclerosis, suggesting a unique regulatory role for IGFBP4 in the plaque dynamics through KLF15/IGFBP4 axis [68]. Thus, IGFBP4 appears to be involved in various processes; nevertheless, its underlying regulatory mechanisms are still elusive.

Our results present novel findings with significance in OSA research, demonstrating a connection between preclinical observations and clinical implications. The literature presents numerous biomarkers linked to OSA, including inflammatory cytokines (e.g., IL-6, TNF-α) [69], oxidative stress markers [70], and metabolic regulators (e.g., leptin) [71]; however, these markers generally lack specificity to be uniquely associated with OSA. Given the established role of IGF pathways in modulating tissue repair, metabolism, and brain function [72], the relationship between IGFBP4 and IGF signaling [68] makes it an appealing candidate to elucidate the systemic effects of OSA. The findings of this study complement our earlier study on OSA patients, which presented increased serum IGFBP4 levels as a biomarker associated with disease severity [37].

Although the simultaneous increase of HIF1α and VEGF as hypoxia biomarkers serves to validate the occurrence of CIH episodes in our PTFE-animals and CoCl_2_-treated HepG2 cells, it also offers mechanistic insights into the hypoxia-induced regulation of IGFBP4. HIF1α is a major transcription factor active in hypoxic settings that regulates adaptive responses like VEGF expression [42,73]. Previous work suggested the possible involvement of HIF1α in regulating IGFBP4 production through direct or indirect binding to the hypoxia response element (HRE) [74,75]. This idea is corroborated by in vitro research demonstrating increased IGFBP1 promoter activity under hypoxic conditions [74,76]. In relation to OSA, the interaction among HIF1α and IGFBP4 may constitute a pivotal axis connecting hypoxic stress to systemic processes involving IGFBP4.

As a hepatic protein, circulating IGFBP4 is primarily produced and regulated by the liver [63]. Under stressful conditions like hypoxia or inflammation, the liver, as the principal source of plasma IGFBP4, responds adaptively to these systemic stimuli. We report a novel observation of an elevated expression of hepatic IGFBP4 mRNA under hypoxia. This finding highlights the liver’s role in the systemic elevation of IGFBP4 during CIH. Moreover, our finding aligns with earlier studies that reported a potential role for the liver in mediating OSA-related comorbidities, including nonalcoholic fatty liver disease (NAFLD) and insulin resistance [77,78,79]. Future work should elucidate the importance of IGFBP4 in this hypoxia-induced pathway and investigate if modulating hepatic IGFBP4 synthesis could relieve some of the negative consequences associated with OSA. Although IGFBP4 has been identified as a hypoxia-associated marker in OSA, its potential involvement in neurocognitive or behavioral impairments remains unclear. Future longitudinal studies should explore whether IGFBP4 plays a mechanistic role in the neurological consequences of OSA, particularly in relation to hypoxia-induced behavioral deficits.

Although this OSA model confirms the value of IGFBP4 as a biomarker for OSA and offers an opportunity to replicate several characteristics of OSA, it lacks several confounding variables such as obesity, ageing, and genetic predispositions that contribute to OSA variability in humans, which is a recognized limitation of our study. A limitation of this study is the lack of polysomnography, a standard method for OSA confirmation. Its unavailability prevented direct assessment of OSA in the animal model. The molecular processes regulating IGFBP4 upon exposure to CIH are not elucidated and require further research. Future studies should consider pathway analyses to explore the nature of HIF1α to IGFBP4 binding and reveal the downstream signaling pathway targeted by CIH-induced IGFBP4 overexpression. In addition to the animal model-based research, longitudinal studies involving a larger cohort of OSA patients are needed to assess the specificity and accuracy of IGFBP4 as a diagnostic and prognostic biomarker for OSA. Validation of a novel biomarker like IGFBP4 may facilitate the creation of customized treatment plans to normalize IGFBP4 levels, consequently leading to an improvement reflected by OSA remission.

## 4. Materials and Methods

All experimental procedures conform to the guidelines and approval of the animal care committee at the Dasman Diabetes Institute. The protocol was approved by the committee and the office of regulatory affairs in Dasman, Kuwait (Protocol Number: RA AM-2023-010).

### 4.1. Tongue Enlargement by Polytetrafluoroethylene Injection

The study included fifty-five male C57BL/6 mice aged 8 to 12 weeks (Jackson Laboratories, Bar Harbor, ME, USA). Due to the tongue volume’s significance in pharyngeal airway space [80], polytetrafluoroethylene (PTFE) was injected at the mouse tongue’s base to induce a significant tongue enlargement. Our approach was based on the protocol reported by Lebek et al. with some modifications, as recommended by our veterinarian. The density of PTFE, a solid substance, is 2.1 g/mL. To produce 100 μL (50% *w*/*v*), 50 mg of PTFE was reconstituted in glycerol (Sigma Aldrich, St. Louis, MO, USA, Burlington, MA, USA). The prepared combination has 24 μL of pure PTFE in 100 μL, and it is designed to increase the volume of the tongue. Greater injection amounts of PTFE were used only in certain mice to achieve the desired bulking. Animals were randomly assigned to either the test group, which received PTFE injection into the tongue (n = 40 mice) (PTFE; 35 μm particle size; Sigma Aldrich), or the control group, comprising n = 15 mice receiving glycerol as the vehicle control injection. Animals were maintained for a total of 8 weeks. To facilitate the long follow-up observation period, we used younger animals with a mean body weight of 24 g.

Before PTFE injection, animals were intraperitoneally injected with meloxicam (5 mg/kg) combined with saline for optimal analgesia. Before the injection, the cages were placed on a heating pad with a cold spot (1/3 cold). Induction of anesthesia was achieved with 5% isoflurane infusion into the anesthesia chamber. Following the induction of anesthesia, the mice were positioned supinely on a surgical plate while maintaining 1–2% isoflurane infusion to keep the animal anaesthetized. The injection procedure was performed under the microscope, where the mouse tongue was manually pulled as far as possible out of the oral cavity to allow access to the base of the tongue. About 100 μL of PTFE was injected in the tongue base at different spots of the dorsal and ventral side of the tongue using a 27-gauge cannula. Post-intervention, animals were kept in a clean chamber to recover with a heat lamp (250 W) to maintain body temperature until animals regained consciousness. Injected mice were monitored for any post-intervention complications (e.g., bleeding into the tongue, infection or excessive tongue enlargement). Animals were also monitored daily for any changes in skin, food intake, movements, and interaction with other littermates. Animals that showed abnormal behavior reflecting suffering were immediately sacrificed.

Control group animals (n = 15) underwent the same procedure using glycerol (i.e., vehicle control). Upon observing anomalous behavior in a mouse, we promptly euthanized the animal (i.e., 4 mice were sacrificed from the PTFE group). All remaining mice (36/15) had no signs of stress or pain and were tracked throughout the entire observation period of 8 weeks.

### 4.2. Monitoring of Daily Activity

We used PROMETHION Core metabolic cage systems (SABLE Systems International, North Las Vegas, NV, USA) to assess the overall activity of the animals and reflect alterations in their physiological and behavioral patterns, and metabolic data analysis was conducted with Promethion Live software (version 23.0.7). Two weeks post-PTFE injection, animals’ general activity was monitored using metabolic cages, comparing PTFE-injected mice with control animals. The system was calibrated in accordance with the manufacturer’s guidelines. A 24-h experiment begins with calibrating gas analyzers (O_2_ and CO_2_) and flow regulators to ensure sub-0.1% measurement accuracy for respiratory quotient calculations. To mitigate acclimatization stress, baseline gas concentrations are monitored, and animals are introduced to the metabolic cages 24 h in advance. To monitor the XYZ-axis movement, mice were individually kept in cages equipped with standardized food hoppers, water bottles with load cells (±0.01 g resolution), and 3D infrared laser matrices (1 cm spatial resolution). To replicate the circadian rhythms of the human body, environmental compartments are pre-programmed to maintain a temperature of 22 °C and 12:12 light–dark cycles. Pull-mode ventilation is employed to sustain a 2000 mL/min airflow per cage during the experiment. This airflow is synchronized with 3-min sampling intervals via the CGF module (i.e., control-gas-flow) to capture energy expenditure, carbon dioxide production (VCO_2_), and oxygen consumption (VO_2_). Food access control modules and crumb-catching containers minimize caching artefacts while ensuring precise intake measurements. The system is also equipped with integrated weighing platforms to facilitate continuous body mass monitoring (±0.01 g). The Promethion Live software unifies data streams from all sensors, and raw data is automatically corrected for chamber-specific drift using baseline values. This configuration permits the simultaneous monitoring of 8 mice while ensuring 97% measurement consistency across enclosures, thereby reducing inter-individual variability in OSA hypoxia response studies.

### 4.3. Open-Field Test

In animal studies, the open-field test serves to evaluate anxiety-like behaviors and locomotor impairments potentially linked to certain chronic conditions. Anxiety levels were indicated by the time spent near walls versus the center. The test was performed in a sound-insulated open-field box (W × L × D = 50 cm × 50 cm × 50 cm) equipped with a digital camera connected to a computer and ran ANY-Maze, a video-tracking system (ANY-Maze, Stoelting Co, Wood Dale, IL, USA). The movement of animals was captured, quantified, and analyzed by ANY-Maze software version 7.4. Before testing, animals were allowed to acclimate for an hour, and each animal was tested for 30 min. The chamber was cleaned thoroughly using 70% ethanol between sessions. The collected data include total distance traveled, number of entries to the central/open zone, and duration of time spent at each zone. The same experimenter performed recordings, and animals were tested randomly.

### 4.4. Novel Object Recognition Test

The novel object recognition (NOR) test is employed in OSA model studies to assess cognitive deficits, particularly hippocampal-dependent recognition memory [81]. The test was performed in a chamber equipped with an overhead movement-tracking digital camera, as described above. ANY-Maze software was used to set up parameters for NOR that involve defining a familiar object zone and a novel object zone in the chamber. Animals are subjected to an initial 10-min training stage, after which they rest for an hour, followed by a 5-min testing phase that first involves introducing a novel object with a similar texture and size to the familiar object to the chamber. The animal is allowed enough time to explore the objects, at least 20 s within 10 min. Animals not exceeding 20 s of exploration time are excluded from the test. The chamber was cleaned thoroughly using 70% ethanol between recordings. The software analyzed the time spent exploring the objects according to the following equation:

Recognition index (RI) = time novel (TN)/(TN + time familiar (TF)), and

Discrimination index (DI) = (TN − TF)/(TN +TF).

### 4.5. Measurement of the Tongue Diameter

Tongue size was measured following sacrifice. This involved extracting the entire tongue without disrupting its structure, as performed for both PTFE and CTRL animals. Extracted tongues were arranged on a scaled sheet and photographed for documentation and further analysis. The following protocol was employed to analyze the size and determine the area of mouse tongue tissue using ImageJ2 (version 2.16, National Institutes of Health, Bethesda, MD, USA). Images were imported into Fiji/ImageJ2 and converted to 8-bit grayscale, whilst using the threshold tool and applying noise reduction enabled the differentiation of the tongue tissue from the background. The total tissue area was quantified with the analyzer particles tool, and the results were converted to metric units using the scale bar reference. The same investigator took all measurements.

### 4.6. Isolation of RNA and Transcription into cDNA

Total RNA was extracted from tissues using TRIzol^®^ (ThermoFisher Scientific, Waltham, MA, USA). After tissue homogenization, RNA was extracted and quantified, and 2 μg of RNA was reverse-transcribed to cDNA using a cDNA kit (ThermoFisher Scientific, Waltham, MA, USA) following manufacturer’s guidelines and stored at 4 °C.

### 4.7. Quantification of Hypoxia Markers

The hypoxia markers HIF1α and VEGF mRNA expression levels were evaluated in tissue samples obtained from PTFE and control animals using qPCR with cDNA on the QuantStudio^TM^ 5 (Applied Biosystems, Waltham, MA, USA). Experiments were conducted with the following parameters: initial incubation at 50 °C for 2 min and polymerase activation at 95 °C for 10 min, followed by 40 cycles at 95 °C for 15 s and 60 °C for 1 min, adhering to the manufacturer’s guidelines. Pre-designed probes from Applied Biosystems were utilized to quantify HIF1α (2405063466) (forward primer 5′ ACCCATTCCTCATCCGTCAA 3′, reverse primer 5′ AATTGAGCGGCCCAAAGTT 3′), KDM6A (2405063466) (forward primer 5′ GTCTTGTGCGGAGATTGGAG 3′, reverse primer 5′ CGCCGCCATTTTCTTTTCCT 3′), and GAPDH (2405063466) (forward 5′ CCCACTCTTCCACCTTCGAT 3′, reverse 5′ CTTGCTCAGTGTCCTTGCTG 3′).

All samples were tested in duplicates, and the mean threshold cycle (Ct) was used for the comparative Ct relative quantification analysis. The average Ct of each target was normalized to the average delta Ct (∆Ct) of the housekeeping gene GAPDH. The calculated ∆Ct was subtracted from the average ∆Ct of the control samples, yielding the delta-delta Ct value (∆∆Ct). The relative expression of each target gene was determined using the 2^−∆∆Ct^ fold change for each target [82].

### 4.8. In Vitro Chemical Induction of Hypoxia

Human hepatoma cells (HepG2) were cultured, expanded, and maintained in T75 cm^2^ polystyrene flasks with Dulbecco’s modified Eagle’s medium (DMEM) supplemented with 10% fetal calf serum, 1 mM sodium pyruvate, 10 mM HEPES, 50 μg/mL penicillin, and 100 μg/mL streptomycin and incubated under 5% CO_2_. Cells were grown to 70% confluence, and on the day of the experiment, cells were treated with CoCl_2_ (150 μM) media or a vehicle-containing media for 24 h.

### 4.9. IGFBPs Quantitative Assays

IGFBPs levels (i.e., IGFBP1 and IGFBP3) were quantified by the Magnetic Luminex Assay kit (R&D Systems Europe, Ltd., Abingdon, UK), and IGFBP4 levels were determined with the mouse insulin-like growth factor protein 4, IGFBP-4 ELISA kit (CUSABIO, code CSB-E07355m, CUBIO innovation center, Houston, TX, USA), following the manufacturer’s protocol.

### 4.10. Data Analysis

Statistical analyses were determined according to the number of animals, and data are presented as the mean ± standard error of the mean (SEM). To evaluate the statistical significance between the PTFE and CTRL groups, we employed an unpaired Student’s *t*-test. For multiple groups, a groups comparison was performed with a one-way ANOVA test, and a post hoc Dunnett test was used to correct for multiple comparisons. GraphPad Prism 10 was employed to conduct all statistical tests to determine significance. Statistical significance was determined by two-sided *p*-values that were less than 0.05.

## 5. Conclusions

In conclusion, our study utilized the PTFE OSA mouse model to validate IGFBP4 as a novel and specific biomarker for OSA, connecting it to our previous clinical findings. The substantial increase in circulating IGFBP4 and hepatic IGFBP4 expression (i.e., in vivo and in vitro) highlights the biological significance of IGFBP4 in CIH-induced disease. This report presents novel findings underscoring the necessity to explore the physiological roles of IGFBP4 to enhance comprehension of its therapeutic potential, hence mitigating the detrimental effects of OSA.

## Data Availability

The original contributions presented in this study are included in the article. Further inquiries can be directed to the corresponding author(s).

## Abbreviations

The following abbreviations are used in this manuscript:

NOR	Novel object recognition
OSA	Obstructive sleep apnea
PTFE	Polytetrafluoroethylene
IGFBPs	IGF-binding proteins
IH	Intermittent hypoxia
CVD	Cardiovascular disease
